# Single-distance, phase-only frequency-domain NIRS for vascular oxygenation and tissue metabolism: a Monte Carlo simulation study

**DOI:** 10.1117/1.JBO.30.S3.S34113

**Published:** 2025-10-16

**Authors:** Muaaz Faiyazuddin, Haiyang Tang, Yiqing Hu, Miles Bartlett, Michael D. Nelson, Hanli Liu

**Affiliations:** a University of Texas at Arlington, Department of Bioengineering, Arlington, Texas, United States; b University of Texas at Arlington, Department of Kinesiology, Arlington, Texas, United States

**Keywords:** frequency-domain near-infrared spectroscopy, absolute quantifications of chromophore concentrations, redox-state of cytochrome c oxidase

## Abstract

**Significance:**

Frequency-domain near-infrared spectroscopy (FD-NIRS) currently enables absolute hemoglobin quantification but requires multidistance measurements of both amplitude attenuation and phase shifts. Notably, existing FD-NIRS approaches have not demonstrated reliable quantification of differential redox-state concentrations of cytochrome c oxidase (${\mathrm{CCO}}_{\text{redox}}$), a critical metabolic marker.

**Aim:**

We aimed to develop a novel optimization-based algorithm for single source-detector (S-D), phase-only FD-NIRS that achieves accurate quantifications of hemoglobin parameters (HbO and Hb) and ${\mathrm{CCO}}_{\text{redox}}$.

**Approach:**

Our computational framework implemented both forward modeling and inverse reconstruction. For the modeling, we first defined chromophore concentration sets (HbO, Hb, ${\mathrm{CCO}}_{\text{redox}}$), followed by calculations of wavelength-dependent optical properties for two- or eight-wavelength configurations. Next, time-domain photon propagation was generated via Monte Carlo (MC) simulations, and FD-NIRS parameters (modulation amplitude, phase) were extracted through Fourier analysis. In the inverse computation, nonlinear optimization with edge-barrier regularization was employed for the recovery of chromophore concentrations. Both the multiseparation method and the single S-D, phase-only algorithm were used to reconstruct chromophore concentrations.

**Results:**

Respective performances evaluated for the two methods were compared through their concentration recovery accuracy. In either the two- or eight-wavelength configuration, our new algorithm outperformed the conventional method for the S-D separations up to 3 cm for all three chromophores. In particular, ${\mathrm{CCO}}_{\text{redox}}$ estimation was improved markedly from a mean relative error of 34.1% with the conventional method to just 5.1% using our algorithm.

**Conclusions:**

These results validate single-separation phase-only FD-NIRS as an accurate method for multichromophore quantification (including ${\mathrm{CCO}}_{\text{redox}}$), enabling simpler, cost-effective systems without compromising metabolic imaging capability. The approach achieves <10% error in hemoglobin quantification while eliminating traditional multidistance requirements.

## 1 Introduction

Near-infrared spectroscopy (NIRS) is a noninvasive optical technique that quantifies tissue chromophores by measuring near-infrared light attenuation through biological tissue. Among various NIRS modalities, frequency-domain NIRS (FD-NIRS) utilizes a radio frequency-modulated light source containing both direct and alternating current (DC and AC) components, generating a photon-density wave that propagates through the measured tissue.^1^^,^^2^ FD-NIRS devices measure attenuations in both DC and AC amplitudes along with phase shifts of this photon-density wave. By combining these measurements, FD-NIRS enables determination of absolute tissue optical properties—specifically, the absorption coefficient ($\mu_{a}$) and reduced scattering coefficient ($\mu_{s}^{\prime }$)—which in turn permit calculation of absolute chromophore concentrations.^1^^,^^2^ Theoretically, accurate measurement of tissue $\mu_{a}$ at multiple wavelengths allows quantification of not only vascular oxyhemoglobin (HbO) and deoxyhemoglobin (Hb) concentrations but also (though more challenging) the differential redox-state concentration of cytochrome c oxidase (CCO).^3^^,^^4^

A widely used approach for quantifying tissue optical properties *in vivo* using FD-NIRS is the slope method, which requires measurements at multiple source-detector (S-D) separations.^1^^,^^2^^,^^5^ This method is based on FD diffusion theory, which demonstrates that in an infinite or a semi-infinite boundary setting, both: (1) the logarithm of the product of AC (or DC) amplitude and S-D separation (ρ), and (2) the phase delay, exhibit linear correlations with ρ.^5^ These linear relationships enable the determination of the absorption coefficient ($\mu_{a}$) and reduced scattering coefficient ($\mu_{s}^{\prime }$) through simple linear regression of the respective slopes.^1^^,^^5^ Although computationally straightforward and experimentally validated, the slope method has notable limitations. First, it necessitates multiple spatial channels for different ρ values, increasing both system complexity and cost. More importantly, although employing multiple S-D separations, the method assumes tissue homogeneity across all measurement locations and therefore cannot provide depth-resolved information.

To address these limitations, alternative FD-NIRS strategies have been developed, such as a single-distance but frequency-sweep approach^6^ and a dual-slope method.^7^^,^^8^ However, these approaches often require more sophisticated hardware (e.g., frequency-sweep device) or rely on additional modeling development.^7^^,^^8^ These constraints limit their generalizability and practical utility for clinical or wearable applications, where simplicity, robustness, and cost-effectiveness are paramount.

In this study, we hypothesized that an optimization-based algorithm could simultaneously estimate chromophore concentrations and tissue scattering parameters using only phase-shift measurements from FD-NIRS at a single S-D separation and modulation frequency. To validate this hypothesis, we employed Monte Carlo (MC) simulations to generate FD-NIRS data with well-controlled optical properties and chromophore concentrations, enabling rigorous evaluation of the algorithm’s performance across diverse parameter conditions. Given its reliance on phase-only FD-NIRS measurements at a single S-D separation, we termed the algorithm ${\mathrm{FD}}_{1\mathrm{SD}\_\mathrm{phase}}$. As a purely computational tool for post-processing, ${\mathrm{FD}}_{1\mathrm{SD}\_\mathrm{phase}}$ is compatible with conventional FD-NIRS hardware and measurement geometries, ensuring broad applicability.

Furthermore, we expanded the capabilities of our ${\mathrm{FD}}_{1\mathrm{SD}\_\mathrm{phase}}$ framework beyond conventional hemoglobin quantification using dual-wavelength approaches. By simulating FD-NIRS phase measurements across eight carefully selected NIR wavelengths,^9^ we demonstrate, as a proof of principle, that ${\mathrm{FD}}_{1\mathrm{SD}\_\mathrm{phase}}$ can estimate the differential redox-state concentration of cytochrome c oxidase (${\mathrm{CCO}}_{\text{redox}}$), defined as the difference between oxidized and reduced CCO (${\mathrm{CCO}}_{\text{redox}}=\mathrm{oxidized}$ [CCO] − reduced [CCO]). Accordingly, ${\mathrm{CCO}}_{\text{redox}}$ serves as a critical marker or characteristic of mitochondrial metabolism.^4^^,^^10^ Although prior studies have quantified changes in ${\mathrm{CCO}}_{\text{redox}}$ relative to baseline using broadband continuous-wave NIRS^10^^,^^11^ or hybrid methods,^12^ absolute ${\mathrm{CCO}}_{\text{redox}}$ quantification remains rare and technically challenging due to its low concentration in tissues and subtle spectral features. Our Monte Carlo simulations reveal that ${\mathrm{FD}}_{1\mathrm{SD}\_\mathrm{phase}}$, when applied to FD-NIRS data at these eight optimized wavelengths, enables robust ${\mathrm{CCO}}_{\text{redox}}$ estimation, particularly at source-detector separations shorter than or equal to 2.5 cm.

Through Monte Carlo simulations, this study validates our hypothesis and demonstrates the feasibility of solving the inverse problem using our approach as a standalone methodology. Although experimental demonstrations and broader clinical applications remain important future directions, the key contribution of this work lies in developing a single S-D separation, single modulation-frequency, and phase-only FD-NIRS technique. This innovation represents a significant step toward simpler, more cost-effective FD-NIRS implementations that could enhance its practicality for clinical and wearable applications.

## 2 Theory

### 2.1 FD Photon Propagation in Scattering Media

In FD-NIRS, a tissue volume is illuminated with intensity-modulated NIR light, and the detected signal at the surface is characterized by both amplitude attenuation and phase delay. These arise due to the combined effects of absorption and scattering within the tissue and are described by the diffusion approximation to the radiative transport equation, valid under the conditions of high scattering and low absorption.^1^^,^^2^

Several analytical solutions to the FD diffusion equation exist, differing primarily in the boundary conditions imposed. The infinite medium model assumes unbounded geometry, whereas the semi-infinite model reflects the presence of a tissue–air interface. Among these, the semi-infinite solution with extrapolated boundary conditions accounts for refractive index mismatch and surface reflections, offering a more realistic representation of experimental geometry.^3^^,^^5^ As such, it has been widely used in FD-NIRS for modeling light propagation in homogeneous tissue volumes. Specifically, Eq. (1) models the phase of the photon-density wave as a function of the tissue’s absorption coefficient $\mu_{a}$, reduced scattering coefficient $\mu_{s}^{\prime }$, source-detector separation ρ, modulation frequency ω, and the speed of light in the medium v  

$$\Phi^{s}=\rho \cdot \sqrt{\frac{\mu_{a}}{2D}}\cdot \sqrt{\sqrt{\left(1+(\frac{\omega }{v\mu_{a}}{)}^{2}\right)}-1}-\arctan(\frac{\rho \cdot \sqrt{\frac{\mu_{a}}{2D}}\cdot \sqrt{\sqrt{\left(1+(\frac{\omega }{v\mu_{a}}{)}^{2}\right)}-1}}{1+\rho \cdot \sqrt{\frac{\mu_{a}}{2D}}\cdot \sqrt{\sqrt{\left(1+(\frac{\omega }{v\mu_{a}}{)}^{2}\right)}+1}}).$$ (1)

In contrast to the slope method, the approach described in this study leverages single-distance, phase-only measurements of FD-NIRS, obtained across multiple wavelengths, to estimate tissue optical properties through a nonlinear optimization strategy. This modeling framework removes the requirement for spatial hardware complexity and enables spectral resolution of individual chromophores.

### 2.2 Spectral Modeling of Tissue Optical Properties

To enable the recovery of physiological parameters from multispectral FD-NIRS measurements, we define the tissue’s optical properties as functions of a small number of wavelength-independent variables, as given below. The absorption coefficient at each wavelength λ is modeled as a linear combination of known extinction coefficients and unknown chromophore concentrations

$$\mu_{a}(\lambda )=\sum_{i}\varepsilon_{i}(\lambda )\cdot [C_{i}]+\mu_{a,\mathrm{water}}(\lambda ),$$ (2)

where $\varepsilon_{i}(\lambda )$ is the molar extinction coefficient of chromophore i∈{HbO,Hb,CCO}, and $\mu_{a,\mathrm{water}}$ accounts for water absorption when applicable. In this study, we do not directly fit $\mu_{a}(\lambda )$ but rather use them as intermediate variables to reconstruct underlying concentrations.

Similarly, the reduced scattering coefficient is modeled using a power-law dependence on wavelength^13^

$$\mu_{s}^{\prime }(\lambda )=a(\frac{\lambda_{0}}{\lambda }{)}^{-b},$$ (3)

where a is the scattering amplitude, b is the scattering power, and $\lambda_{0}$ is a reference wavelength (500 nm in this work).

Equations (2) and (3) associate the wavelength-independent parameters $[C_{i}],a$, and b, with the full spectral profiles of $\mu_{a}(\lambda )$ and $\mu_{s}^{\prime }(\lambda )$. When multiwavelength (e.g., >8) measurements are performed, the parameterization given by Eqs. (2) and (3) is the key to significantly reducing the dimensionality of the inverse problem. Rather than reconstructing a separate pair of $\mu_{a}$ and $\mu_{s}^{\prime }$ for each wavelength, this study reconstructed a compact set of physiologically meaningful parameters (i.e., chromophore concentrations and tissue scattering properties), from which the full optical spectra can also be obtained. In this way, we enabled effective spectral unmixing from a limited number of measurements, including configurations with only two wavelengths for obtaining HbO and Hb or eight wavelengths for estimating HbO, Hb, and ${\mathrm{CCO}}_{\text{redox}}$.

### 2.3 Inverse Estimation with Nonlinear Optimization

The estimation of tissue physiological parameters is considered a nonlinear inverse problem. Given multiwavelength phase measurements at a fixed S-D separation and modulation frequency, the goal of this study was to recover the underlying chromophore concentrations and scattering parameters that minimize the discrepancy between the measured and predicted phase shifts. Given the analytical phase expression described in Eq. (1) and the spectral parameterizations defined by Eqs. (2) and (3), the inverse problem is then formulated as the minimization of a cost function, defined as the sum of squared errors between the modeled and simulated phase values across all the wavelengths selected, as shown in Eq. (4)

$$\mathrm{Error}=\sum_{\lambda }[\varphi_{\mathrm{model}}(\lambda ;\theta )-\varphi_{\mathrm{meas}}(\lambda ){]}^{2}+R(\theta ),$$ (4)

where $\varphi_{\mathrm{model}}(\lambda ;\theta )$ denotes the predicted phase at wavelength λ, computed from the model using the parameter set $\theta =\{[\mathrm{HbO}],[\mathrm{Hb}],[{\mathrm{CCO}}_{redox}],a,b\}$. The $\varphi_{\mathrm{meas}}(\lambda )$ represents the phase shift values obtained from Monte Carlo simulations. The first term in the cost function, Eq. (4), captures the spectral mismatch between the model and data, whereas the second term R(θ) serves as a regularization penalty.

To enforce physiological plausibility and prevent overfitting, a custom edge-barrier regularization scheme is employed. This penalty discourages parameter values from drifting toward the extremes of their admissible range but remains permissive if the data strongly support such values. Although conceptually related to quadratic penalty methods and soft constraint enforcement in constrained optimization, this formulation was specifically developed for concentrations estimation in this study. The regularization function is defined as

$$R(\theta_{j})=\alpha \cdot (\frac{\theta_{j}-c}{h}{)}^{2},\;\mathrm{where}\;c=\frac{\theta_{\mathrm{upper}}+\theta_{\mathrm{lower}}}{2},h=\frac{\theta_{\mathrm{upper}}-\theta_{\mathrm{lower}}}{2}.$$ (5)

The optimization process was constrained by the following bounds: HbO (30 to 90  μM), Hb (5 to 40  μM), CCO (0 to 16  μM), scattering coefficient a (15 to 50), and scattering exponent b (0.5 to 2). These bounds were represented in θ upper and θ lower and then used to determine the auxiliary parameters c and h for the regularization described in Eq. (5). In this way, each parameter is assigned a penalty weight α, allowing differential control over the smoothness and range tightness of the solution. This formulation favors estimates that lie within physiologically relevant bounds while still allowing deviation if justified by the measured data.

Although FD-NIRS systems typically measure both amplitude and phase of the photon-density wave, this study utilizes only the phase component for inverse estimation. The decision is based on both theoretical considerations and empirical evaluation. Amplitude is scaled by the source intensity, which varies across trials and appears as a multiplicative factor in diffusion theory. To account for this, we incorporated a scaling factor β as an additional parameter to be estimated in the optimization framework. This provided flexibility to account for amplitude scaling effects without requiring system calibration or hardware-specific modeling.

To evaluate the scaling factor β, we tested a two-stage optimization strategy: initial phase-only fitting followed by joint phase and amplitude refinement. However, the modeling of β proved problematic, as its magnitude is orders larger than physiological parameters (e.g., chromophore concentrations and scattering coefficients), distorting the optimization landscape and hindering convergence. Simulations showed that the combined approach offered only marginal accuracy gains while increasing complexity and instability. By contrast, phase-only fitting is free from scaling ambiguity and produces robust and consistent results. Accordingly, we formulated a phase-only optimization algorithm that eliminated calibration dependence and enabled a single-distance design.

The cost function [i.e., Eq. (4)] was minimized using the L-BFGS-B algorithm, a quasi-Newton method suitable for bounded nonlinear optimization.^14^ Parameter bounds were derived from literature-reported physiological ranges.^3^^,^^13^ Optimization was performed independently for each measurement point using vectorized spectral fitting, supporting both dual-wavelength and eight-wavelength configurations. Notably, our algorithm demonstrates consistent convergence even when initialized with fixed values that deviate substantially from the ground truth, underscoring its robustness and generalizability.

## 3 Methods

**Fig. 1:**
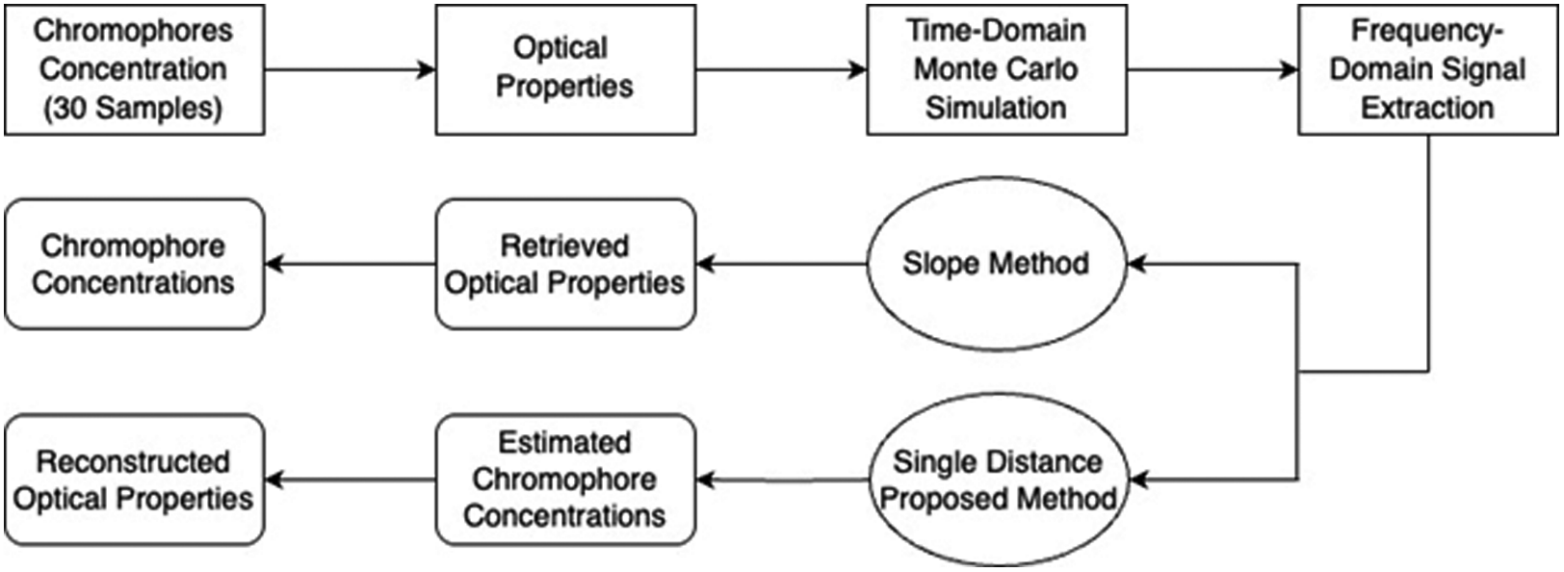
Flowchart to present the simulation and analysis workflow used in this study. It starts from a chosen set of chromophore concentrations (HbO and Hb with or without ${\mathrm{CCO}}_{\text{redox}}$), followed by corresponding $\mu_{a}(\lambda )$ for given wavelengths (either two or eight wavelengths). Then, FD-NIRS parameters (i.e., DC, AC, and phase values) were extracted from the time-domain MC simulations after the Fourier transform. Next, both the slope method and single S-D, phase-only optimization method were used to reconstruct chromophore concentrations.

Figure 1 presents a flowchart summarizing the computer simulation and data analysis workflow. The process begins with a predefined set of chromophore concentrations (HbO and Hb), from which the corresponding $\mu_{a}(\lambda )$ and $\mu_{s}^{\prime }(\lambda )$ spectra are derived. These spectra serve as inputs for time-domain MC simulations. The simulated time-resolved data are then Fourier-transformed to extract FD-NIRS parameters, namely, the DC amplitude, AC amplitude, and phase values. Using these FD-NIRS parameters, we applied two distinct reconstruction approaches: One was the conventional slope method (requiring multiple source-detector separations), and the other was our proposed single S-D, phase-only optimization method. The workflow was repeated 30 times with independently and randomly perturbed chromophore concentrations and scattering parameters within a physiologically meaningful range for each respective parameter. Although not exhaustive of all possible physiological states in living tissues, 30 realizations are sufficient to capture inter-subject variability and provide statistically meaningful error distributions. This choice follows the established practice in simulation-based population studies, where a sample size of n=30 is widely regarded as an acceptable standard for generalizable statistical inference.^15^

### 3.1 Synthetic Optical Property Generation

To evaluate the accuracy and generalizability of the proposed ${\mathrm{FD}}_{1\mathrm{SD}\_\mathrm{phase}}$ inversion framework, we generated controlled synthetic datasets that mimic realistic physiological variability in tissue optical properties. The $\mu_{a}(\lambda )$ value was computed based on representative concentrations of the dominant NIR chromophores: HbO, Hb, and ${\mathrm{CCO}}_{\text{redox}}$. For each of these constituents, a physiological mean concentration was assumed, namely, 60, 25, and 8.5  μM for HbO, Hb, and ${\mathrm{CCO}}_{\text{redox}}$, respectively.^16^ Reported brain tissue values vary across studies. We chose values near the higher end of these ranges to remain physiologically realistic while ensuring a sufficient dynamic range in the simulated signals. The CCO concentration was set to 10% of total hemoglobin, in line with reports that its levels are typically less than one-tenth of hemoglobin concentrations.^17^ Sample-to-sample variation (simulating subject-to-subject) was introduced using random perturbations drawn from a Gaussian distribution with a standard deviation of 10% of the mean. This process was used to generate 30 distinct chromophore profiles, intended to simulate inter-subject or inter-region variability within biological tissues.

Absorption due to water was also included as a background term, scaled by a fixed volume fraction of 75%. For each sample, the total absorption coefficient spectrum was calculated at the selected wavelengths by applying Beer’s law across the perturbed concentration vectors. Reduced scattering coefficients $\mu_{s}^{\prime }(\lambda )$ were modeled using Eq. (3), with base parameters a=24.2 and b=1.611, and a reference wavelength of 500 nm. In analogy to chromophore concentration, the scattering parameters were independently perturbed by 1% across samples to represent subject or anatomical variability in tissue microstructure. This process ensured that both $\mu_{a}$ and $\mu_{s}^{\prime }$ values were unique for each sample, forming a physiologically diverse basis for forward simulation.

First, two wavelength configurations were used in the study. For hemoglobin-only estimation, MC simulations were performed at 690 and 830 nm, which are commonly employed for quantifying HbO and Hb due to their differential absorption profiles. Second, for ${\mathrm{CCO}}_{\text{redox}}$ quantification, an eight-wavelength set was adopted (784, 800, 818, 835, 851, 868, 881, and 894 nm) based on a continuous-wave study.^9^ This spectral configuration has been previously shown to enhance sensitivity to the broad and low-concentration absorption features of ${\mathrm{CCO}}_{\text{redox}}$ while maintaining discrimination from overlapping hemoglobin signals.

### 3.2 Monte Carlo Simulation and FD Signal Construction

Time-domain photon propagation was simulated using the pmcx Monte Carlo engine under homogeneous tissue geometry.^18^ A cuboidal tissue volume (10 cm per side) was modeled for the Monte Carlo simulations, with an isotropic point source positioned at the center of the top surface and detectors placed at radial distances of 2.0, 2.5, 3.0, and 3.5 cm from the source. Each simulation used sample-specific $\mu_{a}(\lambda )$ and $\mu_{s}^{\prime }(\lambda )$ profiles. A fixed anisotropy factor (g=0.85) and refractive index (n=1.37) were applied across all simulations. These parameter choices are standard for brain-like tissues and provide a consistent baseline for comparing the inversion methods. Although g and n may vary slightly across tissue types, their effects on photon migration and boundary reflections are minimal as these variations fall within a narrow physiological range.^3^^,^^19^ Therefore, fixing these values did not compromise the development or validation of the algorithms in this study.

In computational experiments, one NVIDIA A100 GPU (Google Colab cloud computing facility) was employed for the Monte Carlo simulations. Each simulation with $1.2\times 10^{8}$ photons at each wavelength took ∼30  s to complete with a temporal resolution of 10 ps over a 10-ns acquisition window. A total computational time for a chosen set of three concentrations of HbO, Hb, ${\mathrm{CCO}}_{\text{redox}}$ was ∼4  min after running all eight wavelengths. This set of computations yielded 32 time-resolved photon reflectance waveforms (4 separations × 8 wavelengths) for further FD-NIRS analysis. In the entire computational experiments, the synthetically simulated samples (subjects) were 30, so the computational time was ∼2  h (4 min/sub × 30 sub = 120 min.).

FD measurements were derived by applying a discrete Fourier transform to each reflectance at a modulation frequency of 110 MHz. From the complex FFT output, three quantities were extracted: the DC and AC amplitudes (steady and modulated component of optical fluence) and the phase shift (angular delay). These values were assembled into structured matrices, with each row representing a sample and each column corresponding to a wavelength–separation pair. The resulting dataset simulates realistic FD-NIRS measurements and supports both dual-wavelength and multispectral analysis workflows.

### 3.3 Slope Method for Optical Property Estimation

To provide a reference for evaluating the proposed single-separation, phase-only inversion algorithm, the conventional slope method was applied to the frequency-domain synthetic data. For each wavelength, a linear fit was performed using the phase values and $\ln(\rho^{2}\cdot U_{\mathrm{AC}})$ against S-D separation, ρ. The slopes from these two fits were used to compute respective $\mu_{a}$ and $\mu_{s}^{\prime }$ via standard diffusion theory relations.^3^^,^^5^ Once each $\mu_{a}$ value was estimated for each wavelength, chromophore concentrations (i.e., HbO, Hb, with or without ${\mathrm{CCO}}_{\text{redox}}$) were recovered via the inversion of the Beer–Lambert law and the known extinction coefficient matrix. This method was applied consistently to both the two-wavelength and eight-wavelength datasets.

### 3.4 Single-Distance, Phase-Only FD-NIRS Optimization Algorithm

The proposed inversion framework was developed to estimate tissue chromophore concentrations and scattering parameters exclusively from single-distance, phase-only FD-NIRS measurements. For each synthetic dataset, the algorithm was applied independently to phase data from four distinct S-D separations (2.0, 2.5, 3.0, and 3.5 cm, labeled SD1–SD4), yielding four sets of independent parameter estimations per sample (or simulated subject).

In each MC simulation, for the two-wavelength case, the estimated model parameters included (1) HbO, (2) Hb, and (3) wavelength-independent scattering parameters, a and b. When additional ${\mathrm{CCO}}_{\text{redox}}$ concentration needs to be determined, the eight-wavelength configuration was used in the optimization process. These four (or five) parameters were optimized via nonlinear least-squares fitting. Subsequently, the spectral profiles of $\mu_{a}(\lambda )$) and $\mu_{s}^{\prime }(\lambda )$ were reconstructed using the relationships given in Eqs. (2) and (3).

We also evaluated a two-feature (i.e., AC amplitude + phase) optimization approach, extending the phase-only fitting process with a gain-scaling parameter. Comparative analyses showed minimal gains in accuracy from including AC amplitude, which is expected given the two-feature method’s increased vulnerability to initialization errors and local minima, particularly for single-distance fitting conditions. Thus, the phase-only method’s robustness justifies its adoption for single-distance applications. Accordingly, we present the results obtained with phase-only optimization in Sec. 4.

### 3.5 Evaluation Metrics and Comparison Strategy

For either the slope method or our ${\mathrm{FD}}_{1\mathrm{SD}\_\mathrm{phase}}$ algorithm, the estimated chromophore concentrations were compared directly against the known values used during the initial synthetic data generation, which served as the ground truth values. All estimations were performed or calculated using fixed initial parameter values (e.g., HbO and Hb) and uniform optimization settings across samples, without any re-tuning or sample-specific initialization. This consistent configuration in assessing both methods underscores the generalizability of the proposed algorithm across physiologically variable conditions.

Estimation accuracy was assessed using two complementary metrics. The mean absolute error (MAE) was used to quantify the average magnitude of deviation between estimated and ground-truth values, expressed in physical units (micromolar for concentrations and inverse centimeter for optical coefficients). In parallel, the mean relative error (MRE) was computed by normalizing the absolute deviation by the corresponding ground truth value, providing a scale-invariant measure of performance. These metrics were computed independently for each chromophore species, as well as for $\mu_{a}$ and $\mu_{s}^{\prime }$ values at each wavelength.

For our ${\mathrm{FD}}_{1\mathrm{SD}\_\mathrm{phase}}$ strategy, each of the four source-detector separations (SD1 through SD4) was treated as an independent estimation, allowing us to examine the sensitivity of the algorithm’s performance to spatial sampling. The results obtained from the single-distance method were then compared with those from the slope method, both of which were evaluated on the same dataset under identical simulation and modeling conditions. This evaluation framework enabled a direct and comprehensive comparison of estimation fidelity across methods, parameters, and wavelength configurations.

## 4 Results

### 4.1 Hemoglobin Quantification Using Two-Wavelength Configuration

The two-wavelength configuration at 690 and 830 nm was used to assess the accuracy of hemoglobin concentration estimates obtained via the proposed ${\mathrm{FD}}_{1\mathrm{SD}\_\mathrm{phase}}$ method. Estimations were performed independently at each of the four S-D separations (2.0, 2.5, 3.0, and 3.5 cm) and were compared against results from the conventional slope method, which used paired AC amplitude and phase measurements across all separations.

**Fig. 2:**
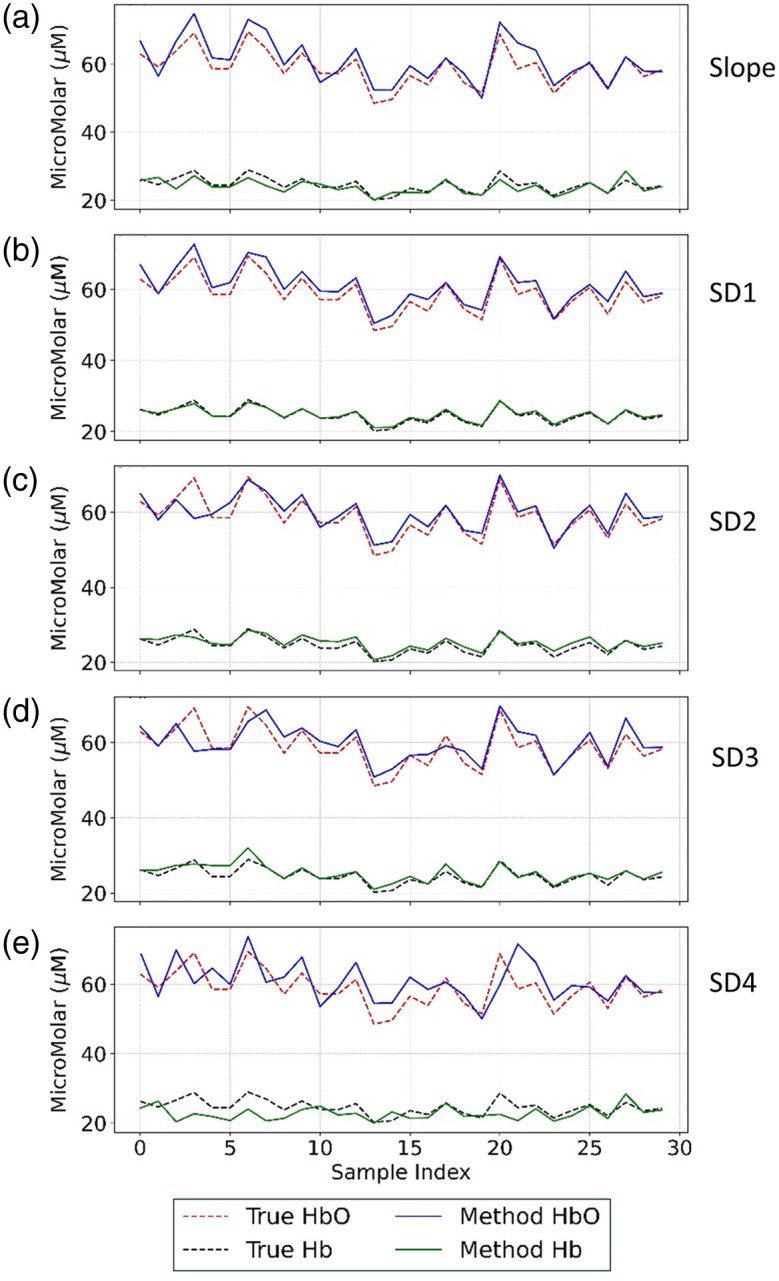
Comparison of true and estimated concentrations of HbO and Hb across 30 synthetic samples using a two-wavelength FD-NIRS configuration (690 and 830 nm). The reconstructed results were obtained using (a) the conventional slope method and ${\mathrm{FD}}_{1\mathrm{SD}\_\mathrm{phase}}$ at (b) SD1 = 2.0 cm, (c) SD2 = 2.5 cm, (d) SD3 = 3.0 cm, and (e) SD4 = 3.5 cm. Dashed lines represent ground-truth concentrations, whereas solid lines denote model estimates. The agreement across samples highlights the performance of each method under the two-wavelength setting.

Figure 2 compares the estimated versus true concentrations of HbO and Hb across all 30 synthetic samples (representing 30 human subjects). For the MC simulations, these synthetic samples were generated with mean concentrations of 60  μM (HbO) and 25  μM (Hb), each perturbed with zero-mean Gaussian noise with a standard deviation equal to 10% of the respective mean concentration to simulate physiological variability. Specifically, Fig. 2(a) displays outcomes from the conventional slope method, whereas Figs. 2(b)–2(e) show the four single-distance variants (SD1-SD4). Visual inspection reveals strong agreement between reconstructed and true concentrations for both chromophores across all samples. However, estimates obtained at the largest source-detector separation (3.5 cm, SD4) exhibit greater fluctuation and systematic bias (particularly for Hb), suggesting reduced measurement reliability at extended distances.

**Fig. 3:**
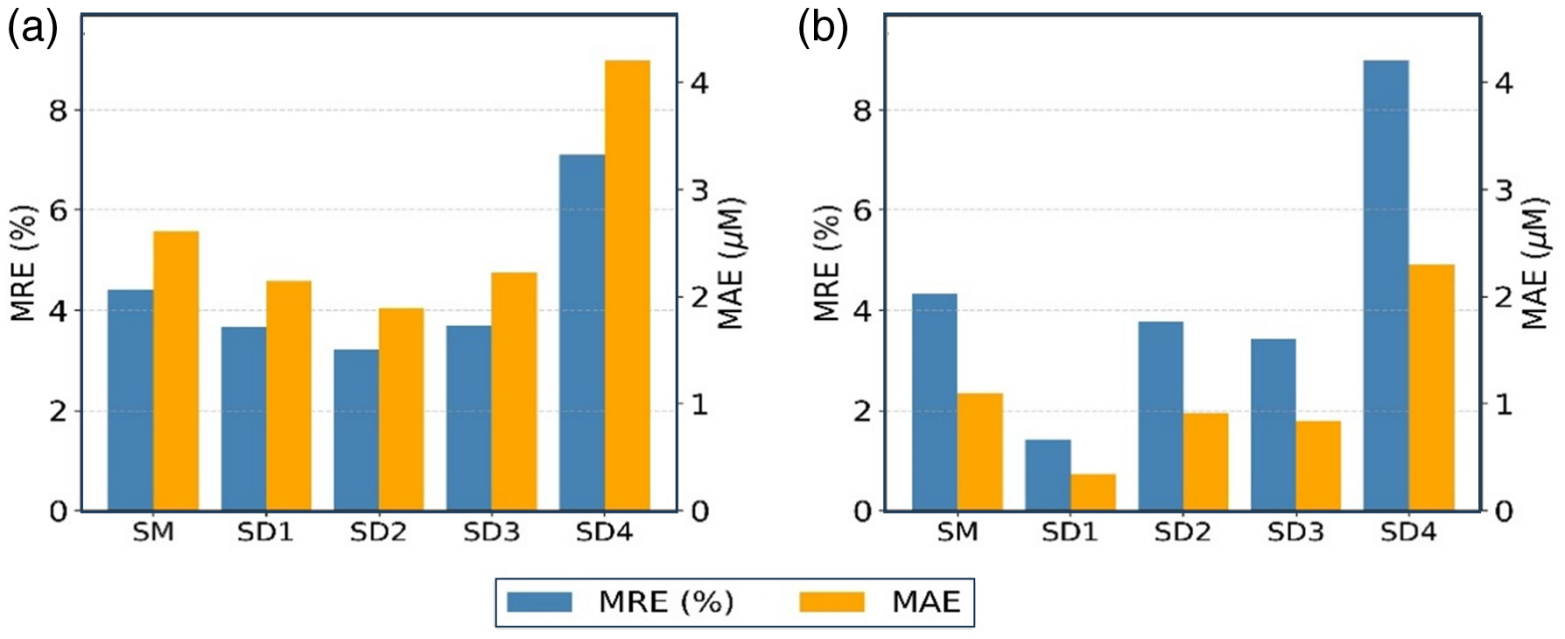
Error comparison for hemoglobin concentration estimates using the two-wavelength configuration (690 and 830 nm). Subplots show mean relative error (MRE, blue bars; left axis) and mean absolute error (MAE, orange bars; right axis) for (a) HbO and (b) Hb across five methods: slope method (SM) and single-distance phase-only inversions at S-D separations of 2.0 cm (SD1), 2.5 cm (SD2), 3.0 cm (SD3), and 3.5 cm (SD4).

The corresponding quantitative errors are summarized in Fig. 3, which presents both MAE in μM and MRE in % for HbO and Hb. Among the single-distance variants, the 2.5-cm S-D separation produced the lowest MAE and MRE for HbO, whereas the most accurate Hb estimation occurred at the 2.0-cm S-D separation, with an MAE of 0.41  μM and an MRE of 1.65%. These results indicate that different S-D separations may yield optimal performance for different chromophores; nevertheless, all single-distance implementations at 2.0 to 3.0 cm achieved better accuracy than the slope method for both hemoglobin species. By contrast, the 3.5 cm separation consistently exhibited higher errors in both MAE and MRE, likely due to diminished phase signal quality and increased susceptibility to modeling inaccuracies at extended S-D distances.

In addition to concentration-level validation, we evaluated the reconstruction accuracy of the optical properties at each wavelength. Tables 1 and 2 report MRE and MAE values in $\mu_{a}$ and $\mu_{s}^{\prime }$, respectively, for both wavelengths. The results demonstrate that the reconstructed values using ${\mathrm{FD}}_{1\mathrm{SD}\_\mathrm{phase}}$ are comparable to those using the slope method across most separations, with MRE values being lower than 5%. This reflects the new algorithm’s ability to reliably reconstruct both light absorption and scattering behavior despite relying solely on single S-D separation and single-phase information.

**Table 1:** MRE and MAE in $\mu_{a}$ estimates at 690 and 830 nm.

Wavelength (nm)	Slope method		SD1 = 2.0 cm		SD2 = 2.5 cm		SD3 = 3.0 cm		SD4 = 3.5 cm	
	MRE (%)	MAE (${\mathrm{cm}}^{-1}$)	MRE (%)	MAE (${\mathrm{cm}}^{-1}$)	MRE (%)	MAE (${\mathrm{cm}}^{-1}$)	MRE (%)	MAE (${\mathrm{cm}}^{-1}$)	MRE (%)	MAE (${\mathrm{cm}}^{-1}$)
690	2.31	0.004	2.07	0.003	3.12	0.005	3.11	0.005	5.14	0.009
830	2.33	0.005	2.99	0.006	2.37	0.005	3.35	0.007	4.50	0.010

**Table 2:** MRE and MAE in $\mu_{s}^{\prime }$ estimates at 690 and 830 nm.

Wavelength (nm)	Slope method		SD1 = 2.0 cm		SD2 = 2.5 cm		SD3 = 3.0 cm		SD4 = 3.5 cm	
	MRE (%)	MAE (${\mathrm{cm}}^{-1}$)	MRE (%)	MAE (${\mathrm{cm}}^{-1}$)	MRE (%)	MAE (${\mathrm{cm}}^{-1}$)	MRE (%)	MAE (${\mathrm{cm}}^{-1}$)	MRE (%)	MAE (${\mathrm{cm}}^{-1}$)
690	5.62	0.81	3.06	0.439	2.78	0.398	3.05	0.438	3.16	0.454
830	2.83	0.301	4.89	0.522	4.72	0.503	4.79	0.511	4.86	0.518

### 4.2 Differential Redox-State CCO Estimation Using Eight-Wavelength Configuration

To evaluate the robustness and spectral generalizability of the proposed inversion framework, we extended the analysis to an eight-wavelength configuration spanning 784 to 894 nm. This range was selected based on established literature demonstrating its effectiveness for quantifying the differential redox-state concentration of CCO alongside hemoglobin species.^9^ The inclusion of ${\mathrm{CCO}}_{\text{redox}}$ adds a layer of complexity because of (1) its relatively low concentration in tissue, (2) shallow absorption features, and (3) an increased risk of crosstalk among chromophore estimates.

Like the reconstruction procedure used in Sec. 4.1, estimates of ${\mathrm{CCO}}_{\text{redox}}$ across 30 MC simulation samples were obtained using both the slope method (which leverages all separations and paired amplitude-phase data) and the single-distance phase-only algorithm applied independently at each of the four source-detector separations. The synthetic samples for ${\mathrm{CCO}}_{\text{redox}}$ were generated with a mean concentration of 8.5μM, with a 10% standard deviation.

At its core, our ${\mathrm{FD}}_{1\mathrm{SD}\_\mathrm{phase}}$ algorithm simultaneously estimated five key parameters—HbO, Hb, ${\mathrm{CCO}}_{\text{redox}}$, and the reduced scattering coefficient parameters (a, b)—through direct optimization using all eight-wavelength MC simulation results. Notably, this approach bypasses the intermediate step of reconstructing wavelength-specific $\mu_{a}(\lambda )$ and $\mu_{s}^{\prime }(\lambda )$ values. In this way, we significantly simplified the optimization problem by (1) reducing the parameter space dimensionality, (2) eliminating spectral reconstruction-related errors, and (3) improving computational efficiency.

**Fig. 4:**
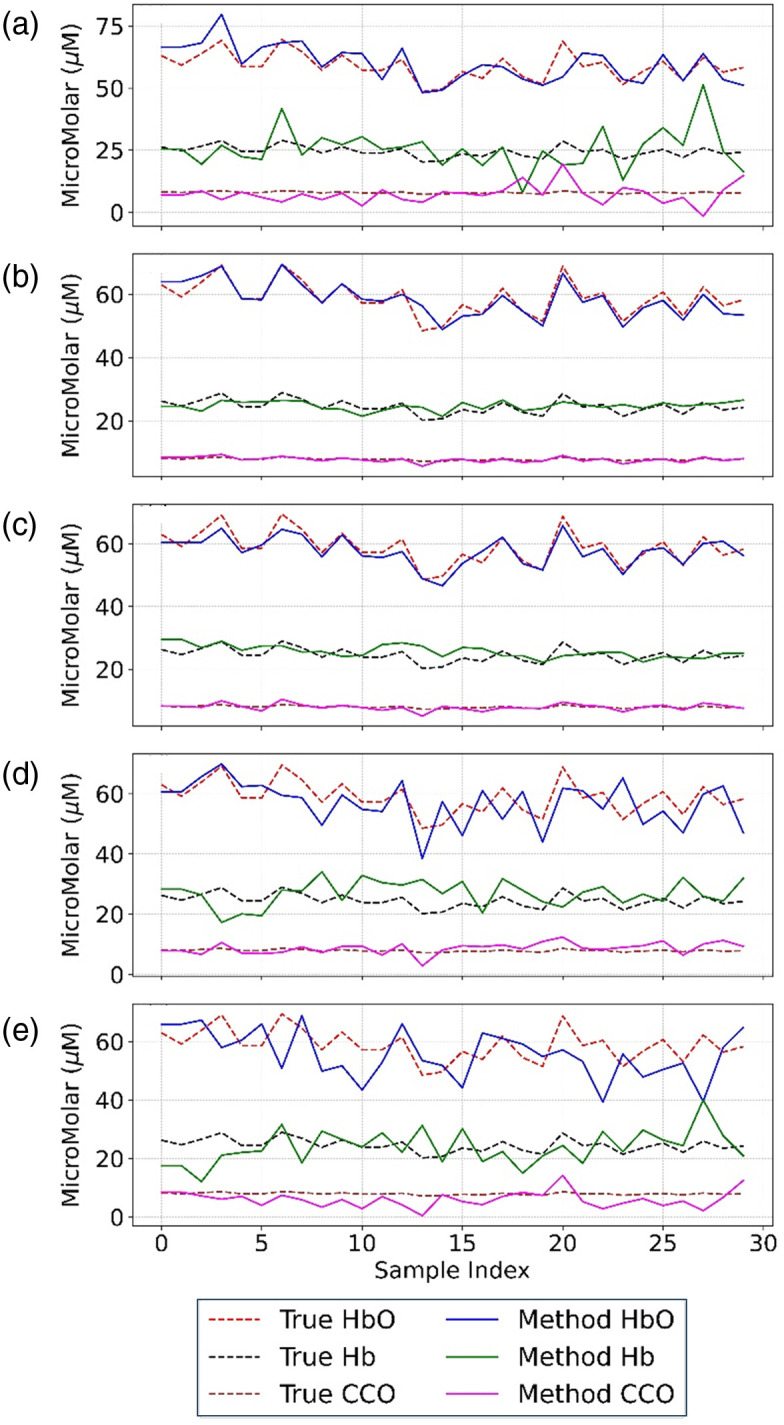
Comparison of true and estimated concentrations of HbO, Hb, and CCO across 30 synthetic samples using an eight-wavelength FD-NIRS configuration (784, 800, 818, 835, 851, 868, 881, and 894 nm). The reconstructed results were obtained using (a) the conventional slope method and ${\mathrm{FD}}_{1\mathrm{SD}\_\mathrm{phase}}$ at (b) SD1 = 2.0 cm, (c) SD2 = 2.5 cm, (d) SD3 = 3.0 cm, and (e) SD4 = 3.5 cm. Dashed lines denote ground-truth concentrations, and solid lines represent estimated values.

Figure 4 presents a comprehensive comparison between estimated and true concentrations for all three chromophores (HbO, Hb, and ${\mathrm{CCO}}_{\text{redox}}$) across the 30 simulated samples. The proposed ${\mathrm{FD}}_{1\mathrm{SD}\_\mathrm{phase}}$ method demonstrates excellent agreement with ground truth values, particularly at shorter S-D separations (2.0 and 2.5 cm). By contrast, the conventional slope method exhibits larger estimation errors, most notably for ${\mathrm{CCO}}_{\text{redox}}$. Key observations include (1) ${\mathrm{FD}}_{1\mathrm{SD}\_\mathrm{phase}}$ maintains strong performance for HbO and Hb up to 3.0 cm separation [Figs. 4(b)–4(d)]), and (2) all chromophore estimates show noticeable accuracy degradation at 3.5 cm, as seen in Fig. 4(e). This performance limitation at larger separations primarily may reflect an increased susceptibility to measuring noise and a greater sensitivity to model mismatch errors.

**Fig. 5:**
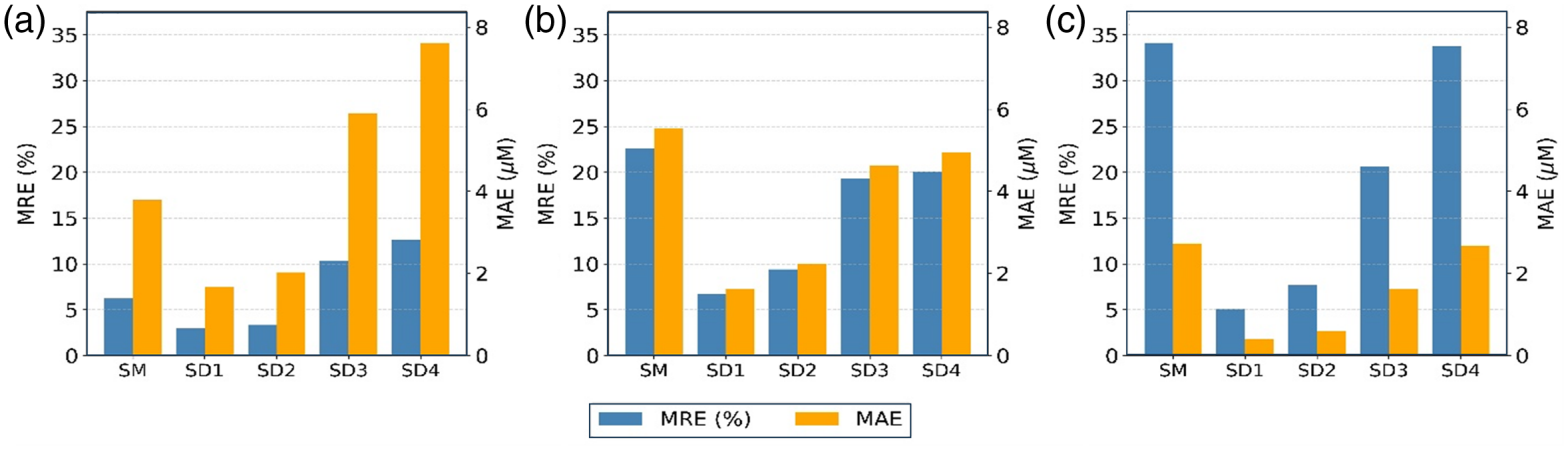
Error comparison for chromophore concentration estimates using the eight-wavelength configuration. Subplots represent comparative results for (a) HbO, (b) Hb, and (c) ${\mathrm{CCO}}_{\text{redox}}$. Each panel shows MRE (blue bars; left axis) and MAE (orange bars; right axis) for the slope method (SM) and single-distance phase-only inversions at S-D separations of 2.0 cm (SD1), 2.5 cm (SD2), 3.0 cm (SD3), and 3.5 cm (SD4).

The quantitative errors for the eight-wavelength configuration are summarized in Fig. 5, showing both MRE and MAE for HbO, Hb, and ${\mathrm{CCO}}_{\text{redox}}$ concentrations. Among the single-distance settings, the 2.0-cm separation yielded the lowest MREs across all three chromophores: 2.95%, 6.71%, and 5.10% for HbO, Hb, and ${\mathrm{CCO}}_{\text{redox}}$, respectively. This separation also achieved the lowest MAEs for HbO (1.68  μM), Hb (1.62  μM), and ${\mathrm{CCO}}_{\text{redox}}$ (0.40  μM). These results highlight the robustness of the proposed single-separation, phase-only method in accurately recovering the ${\mathrm{CCO}}_{\text{redox}}$ concentration, which has low-amplitude within the measurement signals. It implies that ${\mathrm{FD}}_{1\mathrm{SD}\_\mathrm{phase}}$ may have minimal sensitivity to scaling effects and measurement noise.

Figure 5(a) demonstrates that the ${\mathrm{FD}}_{1\mathrm{SD}\_\mathrm{phase}}$ method achieves much lower mean MRE and MAE values at S-D separations equal to or less than 2.5 cm. Moreover, Figs. 5(b)–5(c) reveal that ${\mathrm{FD}}_{1\mathrm{SD}\_\mathrm{phase}}$ consistently outperforms the slope method in both Hb and ${\mathrm{CCO}}_{\text{redox}}$ estimation across all separations, with particularly pronounced improvements at 2.0 to 3.0 cm (SD1-SD3). The most substantial enhancement by our new method was observed for ${\mathrm{CCO}}_{\text{redox}}$, where ${\mathrm{FD}}_{1\mathrm{SD}\_\mathrm{phase}}$ reduced estimation errors remarkably compared with the slope method. The excellent performance of ${\mathrm{FD}}_{1\mathrm{SD}\_\mathrm{phase}}$ remained consistent up to SD2 (S-D separation = 2.5 cm), whereas estimations at 3.0 and 3.5 cm exhibited greater variability and error, likely due to lower phase signal quality and increased sensitivity to model inaccuracies at larger separations. These findings reinforce that the single-separation, phase-only inversion strategy enables high-fidelity quantification of chromophores, including the metabolically critical ${\mathrm{CCO}}_{\text{redox}}$, without reliance on multiple distances or amplitude measurements.

To assess the quality of spectral reconstruction beyond chromophore-level summaries, we evaluated wavelength-wise errors in $\mu_{a}(\lambda )$ and $\mu_{s}^{\prime }$. Tables 3 and 4 report MRE and MAE values for both $\mu_{a}(\lambda )$ and $\mu_{s}^{\prime }$, respectively, at each wavelength for both methods. The results from both tables reaffirm the patterns seen in concentration-level comparisons. The 2.0 to 3.0 cm configurations yield low spectral errors, whereas MRE and MAE values rise substantially at longer separations (>3  cm).

**Table 3:** MRE and MAE in $\mu_{a}$ estimates at each of the eight wavelengths.

Wave-length (nm)	Slope method		SD1 = 2.0 cm		SD2 = 2.5 cm		SD3 = 3.0 cm		SD4 = 3.5 cm	
	MRE (%)	MAE (${\mathrm{cm}}^{-1}$)	MRE (%)	MAE (${\mathrm{cm}}^{-1}$)	MRE (%)	MAE (${\mathrm{cm}}^{-1}$)	MRE (%)	MAE (${\mathrm{cm}}^{-1}$)	MRE (%)	MAE (${\mathrm{cm}}^{-1}$)
784	1.63	0.004	1.66	0.004	2.03	0.004	5.79	0.012	8.38	0.019
800	1.84	0.004	1.65	0.004	1.75	0.004	5.28	0.011	8.91	0.02
818	2.36	0.006	1.70	0.004	1.74	0.004	5.27	0.012	9.01	0.022
835	1.92	0.005	1.66	0.004	1.71	0.005	5.14	0.013	8.51	0.023
851	2.24	0.006	1.67	0.005	1.75	0.005	5.19	0.014	8.36	0.024
868	2.20	0.006	1.69	0.005	1.82	0.005	5.24	0.015	8.23	0.024
881	1.67	0.005	1.70	0.005	1.88	0.006	5.26	0.015	8.15	0.024
894	2.09	0.006	1.70	0.005	1.93	0.006	5.29	0.015	8.09	0.025

**Table 4:** MRE and MAE in $\mu_{s}^{\prime }$ estimates across eight wavelengths.

Wave-length (nm)	Slope method		SD1 = 2.0 cm		SD2 = 2.5 cm		SD3 = 3.0 cm		SD4 = 3.5 cm	
	MRE (%)	MAE (${\mathrm{cm}}^{-1}$)	MRE (%)	MAE (${\mathrm{cm}}^{-1}$)	MRE (%)	MAE (${\mathrm{cm}}^{-1}$)	MRE (%)	MAE (${\mathrm{cm}}^{-1}$)	MRE (%)	MAE (${\mathrm{cm}}^{-1}$)
784	3.51	0.412	3.78	0.441	3.41	0.398	5.63	0.657	5.83	0.683
800	2.62	0.297	3.91	0.442	3.36	0.379	5.57	0.631	5.53	0.627
818	4.60	0.505	3.99	0.436	3.40	0.371	5.85	0.639	5.89	0.645
835	3.87	0.411	4.23	0.447	3.61	0.382	6.04	0.641	5.88	0.622
851	3.13	0.321	4.50	0.461	3.96	0.406	6.48	0.664	6.30	0.645
868	4.20	0.419	4.32	0.43	3.69	0.367	6.42	0.64	6.43	0.643
881	4.57	0.444	4.69	0.455	4.00	0.388	6.75	0.656	6.41	0.625
894	3.81	0.362	4.86	0.46	4.22	0.4	6.95	0.658	7.35	0.699

## 5 Discussion

### 5.1 Reconstruction of Chromophore Concentrations Using FD_1SD_phase_

The results of this study demonstrate that the proposed single-distance, phase-only optimization inversion algorithm for FD-NIRS achieves accurate and robust estimation of chromophore concentrations, outperforming the conventional slope method across multiple evaluation metrics. Across both the two-wavelength and eight-wavelength configurations, our novel method consistently produced lower or comparable mean absolute and relative errors for HbO and Hb concentrations while uniquely enabling the reliable quantification of differential redox-state concentration of CCO, a chromophore that is notoriously difficult to recover with FD-NIRS.

The excellent performance of ${\mathrm{FD}}_{1\mathrm{SD}\_\mathrm{phase}}$ held across a range of separations, with optimal results typically obtained at or shorter than S-D separations of 2.5 cm. Even when estimation was repeated independently at different separations (SD1 to SD4), the algorithm exhibited strong generalizability and multiseparation consistency, with stable performance across varying distances and simulation conditions. These findings suggest that the method is not only accurate but also robust to spatial sampling variation, which is particularly valuable for clinical or wearable FD-NIRS systems with constrained hardware configurations.

Compared with the slope method, which depends on paired AC amplitude and phase measurements across multiple distances, the ${\mathrm{FD}}_{1\mathrm{SD}\_\mathrm{phase}}$ method dramatically reduces system complexity and channel requirements. This simplification could enable broader adoption of FD-NIRS in compact or cost-sensitive platforms. Moreover, although the algorithm is not designed to achieve formal depth resolution, its ability to isolate absorption and scattering effects from a single measurement geometry suggests that ${\mathrm{FD}}_{1\mathrm{SD}\_\mathrm{phase}}$ may be well-suited for integration with tomographic or multilayer reconstruction strategies, potentially enabling hybrid depth-sensitive implementations.

To ensure a fair evaluation of the slope method, we also analyzed an alternative configuration using only three source-detector separations, excluding the largest separation (3.5 cm) due to its relatively poor phase quality under high scattering conditions. However, the resulting estimation accuracy showed no meaningful advantage over the standard four-distance configuration (data not shown). In some cases, the error for one wavelength or chromophore slightly improved, but this was offset by worse performance at other wavelengths. This pattern held for both two-wavelength and eight-wavelength cases, reinforcing that removing a noisy channel does not consistently benefit the slope method.

The superior performance of ${\mathrm{FD}}_{1\mathrm{SD}\_\mathrm{phase}}$ stems from its elimination of the intermediate spectral reconstruction step, bypassing the need to calculate wavelength-specific $\mu_{a}(\lambda )$ and $\mu_{s}^{\prime }(\lambda )$ values. This innovative approach achieves significant dimensionality reduction in the optimization problem by directly estimating the five target parameters (HbO, Hb, ${\mathrm{CCO}}_{\text{redox}}$, and scattering coefficients a and b), instead of 16 parameters in the eight-wavelength case, from raw phase data. However, the method’s effectiveness is currently limited to hemoglobin-containing tissues or phantoms as its parameterization specifically accounts for hemoglobin’s characteristic absorption features.

### 5.2 Reconstruction of Optical Properties Using FD_1SD_phase_

The ${\mathrm{FD}}_{1\mathrm{SD}\_\mathrm{phase}}$ method also achieved strong agreement in the reconstruction of wavelength-resolved optical coefficients (i.e., $\mu_{a}$ and $\mu_{s}^{\prime }$), following accurate quantification of hemoglobin-only or multichromophore estimations, as demonstrated in Tables 1–4. Estimates of $\mu_{a}$ were consistently accurate across all wavelengths, even in the more challenging broadband case. In particular, the reconstructed values of $\mu_{s}^{\prime }$ were slightly overestimated across conditions, typically within a 5–8% MRE across all four source-detector separations. This bias likely arises from the functional form of the power-law model used to parameterize $\mu_{s}^{\prime }$, where both parameters a and b contribute simultaneously to the spectral profile. Because these two parameters can partially compensate for each other during optimization, the regularization penalty may slightly favor a higher scattering parameter a, leading to a slight overestimation of $\mu_{s}^{\prime }$. Despite this small bias, the recoveries of the absorption spectra and chromophore concentrations were robust and remained unaffected by any such trends.

### 5.3 Key Contribution of the Developed Method, FD_1SD_phase_

The successful reconstruction of ${\mathrm{CCO}}_{\text{redox}}$ provides strong evidence of ${\mathrm{FD}}_{1\mathrm{SD}\_\mathrm{phase}}$’s methodological advantages. Notably, although the slope method failed to achieve reliable ${\mathrm{CCO}}_{\text{redox}}$ estimation (showing >30% relative error) even when using an optimized wavelength set,^9^ ${\mathrm{FD}}_{1\mathrm{SD}\_\mathrm{phase}}$ reduced this error to <10%. This level of accuracy represents a significant advancement in FD-NIRS quantification as such precision for ${\mathrm{CCO}}_{\text{redox}}$ measurement has rarely been demonstrated by FD-NIRS. These results establish that, with proper modeling, single-distance, phase-only FD-NIRS can reliably quantify mitochondrial metabolism markers without requiring multidistance or multifrequency measurements. This breakthrough enables reliable tracking of cellular energy metabolism using practical, single-distance FD-NIRS measurements.

### 5.4 Limitations of this Study

Despite these promising results, several limitations warrant discussion. First, the algorithm is presently constrained to hemoglobin-dominant tissues because it requires hemoglobin’s spectral signature for the phase-based optimization. Second, the present study is based exclusively on MC-simulated data with a limited range of chromophore concentrations. Although this provides a rigorous and controllable test environment, experimental validation using actual measurements in tissue-mimicking phantoms or biological tissue will be essential to confirm translational feasibility. Third, the method assumes a homogeneous tissue volume and does not currently account for layered structures or heterogeneous scattering. Last, although the algorithm demonstrated robustness to initialization and noise, further optimization of regularization strategies may improve performance, particularly for weakly absorbing chromophores or lower signal-to-noise conditions.

## 6 Conclusion

This work presents a computationally efficient and experimentally scalable framework for quantitative FD-NIRS using single-distance, single-frequency, phase-only measurements. Using MC-generated synthetic data, we demonstrate that phase data from a single source-detector pair can enable accurate recovery of both chromophore concentrations and tissue scattering properties. Our method outperforms the conventional slope method in hemoglobin-only quantification and multichromophore assessment, including the differential redox-state concentration of CCO, a long-standing challenge in FD-based systems. The algorithm’s success stems from its innovative dimensionality reduction approach, which directly estimates chromophore concentrations while bypassing intermediate spectral reconstruction steps. Although currently validated through *in silico* studies, the results demonstrate the robustness against measurement noise and the scalability to diverse chromophore targets, leading to strong potential for clinical translations.

Future efforts include experimental validation, extension to heterogeneous or layered tissues, and integration with tomographic frameworks to enable depth-sensitive analysis. Collectively, this study establishes a new paradigm for developing simplified yet spectrally comprehensive FD-NIRS systems capable of absolute quantification of both vascular (HbO/Hb) and metabolic (${\mathrm{CCO}}_{\text{redox}}$) markers.

## Data Availability

The data presented in this study are available upon request from the corresponding author.
